# Age-Related Macular Degeneration: Insights into Inflammatory Genes

**DOI:** 10.1155/2014/582842

**Published:** 2014-11-12

**Authors:** Raffaella Cascella, Michele Ragazzo, Claudia Strafella, Filippo Missiroli, Paola Borgiani, Francesco Angelucci, Luigi Tonino Marsella, Andrea Cusumano, Giuseppe Novelli, Federico Ricci, Emiliano Giardina

**Affiliations:** ^1^Department of Biomedicine and Prevention, School of Medicine, University of Rome “Tor Vergata”, Via Montpellier 1, 00133 Rome, Italy; ^2^UOSD Retinal Pathology PTV Foundation “Policlinico Tor Vergata”, Viale Oxford 81, 00133 Rome, Italy; ^3^Molecular Genetics Laboratory UILDM, Santa Lucia Foundation, Via Ardeatina 354, 00142 Rome, Italy

## Abstract

Age-related macular degeneration (AMD) is a progressive neurodegenerative disease that affects approximately 8.7% of elderly people worldwide (>55 years old). AMD is characterized by a multifactorial aetiology that involves several genetic and environmental risk factors (genes, ageing, smoking, family history, dietary habits, oxidative stress, and hypertension). In particular, ageing and cigarette smoking (including oxidative compounds and reactive oxygen species) have been shown to significantly increase susceptibility to the disease. Furthermore, different genes (*CFH, CFI, C2, C3, IL-6, IL-8,* and *ARMS2*) that play a crucial role in the inflammatory pathway have been associated with AMD risk. Several genetic and molecular studies have indicated the participation of inflammatory molecules (cytokines and chemokines), immune cells (macrophages), and complement proteins in the development and progression of the disease. Taking into consideration the genetic and molecular background, this review highlights the genetic role of inflammatory genes involved in AMD pathogenesis and progression.

## 1. Introduction

Age-related macular degeneration (AMD, OMIM** #**610149) is a progressive neurodegenerative and multifactorial disease that impairs the visual field [1, 2]. Its clinical symptoms include a gradual loss of central vision, the distortion of images and straight lines, and the presence of blurry and dark areas in the central vision. Consequently AMD substantially impacts the lifestyle of patients by compromising everyday activities, such as reading and driving [3–5].

AMD affects approximately 8.7% of the elderly population worldwide (>55 years old), especially in developed countries. The number of AMD cases is expected to increase to 196 million in 2020 and to 288 million in 2040 [6]. To date, more than 1 and 10 million people suffer from this ocular disease in Italy and the USA, respectively [7, 8].

The disease affects the small central portion of the retina, known as the* macula lutea*, which is essential for the visualization of fine details and image resolution. In the* macula lutea*, the most common hallmarks of this disease can be recognized in the form of* drusen* (aggregates of extracellular material) and the growth of choroidal vessels (choroidal neovascularization) [9, 10]. Excess of drusen and neovascularization results in chronic changes of the macula and in particular of the retinal pigment epithelium (RPE), choriocapillaries (CC), photoreceptors (rods and cones), and Bruch's membrane (BrM) [11–14]. Two forms of AMD can be distinguished based on anatomic abnormalities: atrophic (dry or nonexudative form) and neovascular or exudative (wet form) [15]. The first, which is known as geographic atrophy, is characterized by the progressive accumulation of drusen between the RPE and CC. Excessive drusen between the RPE and CC hampers the transport of oxygen and nutrients, which degenerates the RPE and photoreceptor system. The dry form may also progress to the more aggressive wet form of AMD, which is characterized by choroidal neovascularization. The process of angiogenesis leads to the formation of very fragile blood vessels, which are responsible for bleeding and the disruption of RPE cells [16, 17].

AMD is a multifactorial disease that involves a continuous interaction between genetic and environmental factors [18]. Among environmental factors, ageing and cigarette smoking significantly contribute to an increase in the AMD risk [19, 20]. In particular, the disease prevalence increased with age and the loss of rod photoreceptors (approximately 30%), which is the result of ageing and thus acts as a joint cause of AMD development [21].

Several studies demonstrated that the AMD risk odds ratio (OR) varies from 1 (in 55–69-year-old people) to 4.42–8.70 (in 70–79-year-old people) and up to 18.8–32.3 (in 80–86-year-old people) [6]. Cigarette smoke contains a high number of toxic substances, which contribute to atherosclerosis, endothelial dysregulation, and angiogenesis. The presence of oxidative compounds in cigarettes is associated with increased reactive oxygen species formation (ROS) and thereby with oxidative damage at the RPE cell level [22–26]. In addition, dietary habits may contribute to disease progression [27]. In fact, dietary supplementation with vitamins C, E, B6, and B12, lutein, zeaxanthin, and zinc has been shown to slow the progression of macular degeneration toward more severe atrophic and/or neovascular forms [28–34].

Concerning the genetic picture of AMD, concordance studies of twins described hereditability as one of the main genetic risk factors for the disease. In fact, the familiarity was estimated to be at least 11% in the presence of one affected first-relative; however, the AMD risk was proven to increase 2.4-fold compared to families without the disease [35–39]. Moreover, a number of studies performed between 2005 and 2007 highlighted* ARMS2* and* CFH* as the major susceptibility loci of the disease, which can cover 50–60% of the AMD genetic picture [40–47]. Genome-wide association studies (GWAS) successively identified common risk variants localized in 17 candidate genes (Table 1) that are potentially involved in the development and progression of the disease [48].

The genes associated with AMD play different roles, but the majority have been demonstrated to serve a function in the inflammatory pathway (Table 2). The knowledge of this association suggested the involvement of inflammatory components in the aetiopathogenesis of the disease. In this context, molecular studies reported the presence of several immunologic and inflammatory molecules in drusen that play a pivotal role in disease progression [49–52].

The aim of this review is to underline the genetic role of inflammatory genes associated with AMD pathogenesis.

## 2. The Role of Inflammation in AMD Pathogenesis

Inflammation is the first biological response to infection with pathogens (bacteria, virus, and fungus) or irritant compounds, and this response represents a protective attempt to remove the injurious stimuli from the organism [53]. The physiological role of inflammation is to preserve tissue homeostasis and restore tissue functionality; nevertheless, this process is also known to be involved in the pathophysiology of different chronic diseases (atherosclerosis, psoriasis, arthritis, inflammatory bowel disease, and neurodegenerative diseases) [54–58].

Of the five common tissue responses that characterize inflammation (*rubor, tumor, calor, dolor,* and* functio laesa*), immunity (innate and adaptive) operates as the sixth actor in the inflammatory scenery [59]. In this context, inflammatory mediators work in tandem with immune cells to regulate acute and chronic inflammation and trigger tissue injury, oxidative stress, extracellular matrix remodelling, angiogenesis, and fibrosis in damaged tissues [60].

The recognition of pathogens and toxins by pattern recognition receptors (PRRs) initiates the innate immune response. The activation of PRRs is considered to be the first step of the inflammatory response [61]. PRR molecules can detect specific* non-self*-structures and proteins of pathogens, which are referred to as pathogen associated molecular patterns (PAMPs). Some PRRs form a multiprotein complex, known as “inflammasomes,” and are involved in the maturation of proinflammatory cytokines [62–64]. The generation of a very large number of antigen receptors (T-cell receptors and immunoglobulins) able to recognize specific molecular structures of the microorganisms encountered by the host activates adaptive immunity. This specialized immunity also requires the participation of specific cytokines. Both innate and adaptive immunity induce the differentiation of effector cells (Natural-Killer cells, antibodies, and cytotoxic lymphocytes) that are responsible for the suppression of the microorganism [59].

The excessive stimulation of the inflammatory and immune responses may result in the development of several degenerative diseases, especially in tissues exposed to elevated oxidative stress and metabolic activity [54, 58, 65, 66]. In this context, the macular retina represents an excellent example of such tissues. In fact, the retina is characterized by different energy-dependent mechanisms that involve high rates of oxygen consumption and mitochondrial oxidative pathways (phototransduction, neurotransmitter utilization, and protein/organelle transport) [67]. Defects in eye metabolism and oxidative stress can lead to neuronal degeneration and retinopathy. AMD is one of the most sight-threatening diseases that results in the anatomic alteration of the macula and persistent activation of inflammatory and immune mechanisms [68]. Harman suggested that AMD could be the result of disequilibrium between stress oxidative damage and repair processes, which suggests that retinal inflammatory processes may contribute to AMD pathogenesis [69]. Several associated genetic studies have implicated inflammatory molecules in the development and progression of the disease.

## 3. Genes Mediating the Inflammatory Process in AMD

### 3.1. Cytokines and Chemokines

Cytokines consist of soluble proteins, peptides, and glycoproteins that regulate many biological processes, including the inflammatory and immune responses. The cytokines that are produced depend on the pro- or anti-inflammatory response (IL-1, IL-8, and TNF-*α* or IL-4, IL-10, and IL-11, resp.). In physiological conditions, the synthesis of both types of cytokines is finely regulated and balanced. Conversely, the deregulation or abnormal production of pro- and anti-inflammatory cytokines represents several inflammatory diseases, autoimmune diseases, or immune deficiency syndromes [70, 71].

Different cytokine families can be distinguished (interleukins, interferons, and tumour necrosis factor). In particular, the interleukins (ILs) are a heterogeneous class of cytokines involved in the activation of T lymphocytes, B lymphocytes, and macrophages. To date, approximately 40 ILs have been characterized based on their structures and functions. Interestingly, genetic polymorphisms in different IL genes (such as* IL-6* and* IL-8*) are known to modify the transcription and production of ILs. Some of them have been associated with AMD pathogenesis, as described in the following section [72–74].

The gene that encodes IL-1 is mapped on chromosome 2q14. Different signals initiate interleukin-1 (IL-1) synthesis (i.e., microbial products), and this cytokine is involved in many inflammatory processes. In fact, IL-1 often acts synergistically with TNF as a proinflammatory molecule, inducing the production of IL-6 and IL-8. IL-6 (its corresponding gene is located on locus 7p15.3) plays a role in inflammation; it mainly stimulates the synthesis of acute phase proteins (like C-reactive protein, complement proteins) in hepatocytes [75, 76].

IL-8 is a member of the CXC chemokine family and plays a role in the chemotaxis of neutrophils and lymphocytes to the site of inflammation. The* IL-8* gene is located on chromosome 4q12-q13. IL-8 protein acts as a mediator molecule in the interaction between two cell-surface G protein-coupled receptors (CXCR1 and CXCR2), and it is also recognized as a primary mediator of angiogenesis [77, 78]. Given its functions, IL-8 plays a pivotal role in the progression of advanced cancer, including angiogenesis, tumour growth, and metastasis. Moreover, IL-8, which is an important mediator of angiogenesis, contributes to plaque formation in human coronary atherosclerosis [79–81].

Given its role in inflammatory mechanisms,* IL-8* may represent a potential candidate gene involved in AMD progression [80]. Several studies have associated a number of* IL-8* polymorphisms with AMD in Asiatic and North European populations. Concerning the Italian population, Ricci et al. performed a genotyping analysis via real-time PCR (TaqMan chemistry) to demonstrate the association of rs2227306 (C/T, intronic SNP in* IL-8* gene) with AMD. The statistical analysis was performed on 721 cases and 660 healthy subjects and reported a significant *P value* of 4.15∗10^−5^ and an OR of 1.39 (95% CI = 1.19–1.62) for the T allele. The entire* IL-8 *region was sequenced to determine the allele architecture of the gene, identifying three AMD-associated SNPs (rs2227346 C/T, rs1126647 A/T, and rs4073 A/T) and one susceptibility haplotype (A-T-T-T, *P value* = 2.8∗10^−9^, OR = 1.68, 95% CI = 1.43–1.97). The potential functional role of* IL-8* in AMD and the correlation between the associated haplotype and its gene expression were further evaluated using mRNA expression analysis. However, the gene expression profile did not vary by genotype class or associated haplotype [82].

Another class of proinflammatory molecules that is potentially linked to AMD pathogenesis is the chemokines, which drive the migration of white blood cells to infected or damaged tissues in response to external stimuli. Chemokines are classified into four main subfamilies according to the amino acidic structure, namely, CXC, CC, C, and CX3C [83]. The genes that encode chemokines are generally clustered. In particular, two clusters that code for the main chemokine subfamilies (CXC and CC) are located on chromosome 4q12-21 and chromosome 17q11.2-q12, respectively. The CC gene cluster contains 16 genes, and 4 of them are paralogous (*CCL3-CCL3L* and* CCL4-CCL4L*) [84]. The* CCL3* and* CCL4* genes further contain specific genomic regions, which are referred to as copy number variations (CNVs), and these regions confer an extensive genetic complexity and interindividual variability to the responsive chemokine molecules. The presence of CNVs in the chemokine-coding regions may be linked to different clinical phenotypes of disease [85, 86].

CC chemokine receptor-3 gene (*CCR3*) is located on the 3p21.3 chromosome and codes for the homonymous receptor (CCR3). It is present on the surface of many cells and plays an important role in angiogenesis, particularly in the activation/recruitment of eosinophils and macrophages to the site of inflammation. A genotyping study of Indian subjects identified rs3091250 in the* CCR3* gene as being significantly associated with AMD and the homozygous TT genotype as a potential risk marker [85].

### 3.2. Genes of Complement System

The complement system consisted of 40 proteins that normally circulate as inactive precursors (proproteins) in the blood and are activated by protease cleavage. The complement system plays a key role in host defence, and three mechanisms can activate the immune response:

1. the classical pathway, which proceeds via antibody-antigen complex activity,
2. the alternative pathway, which identifies the host cell or microbial proteins,
3. the lectin pathway, which recognizes polysaccharide residues on the microorganism surface [87, 88].

The deregulation and dysfunction of complement pathways can change the inflammatory response and are implicated in different diseases, including systemic lupus erythematosus, susceptibility to pyogenic infections, and AMD [59, 89]. Molecular studies have identified many complement proteins in drusen, which are known to be the first clinical sign of the disease. In addition, genetic studies identified specific polymorphisms in complement genes (*CFH*,* C3*,* C2*,* CFI*, and* CFB*) that have been associated with the risk of AMD development and progression [90, 91].

In 2005, the* CFH* gene (chromosome 1q31) was strongly associated with a higher susceptibility to AMD development. CFH (complement factor H) acts as an inhibitor of the adaptive immune response, interacting with the C3 protein and thereby impeding the activation of the complement pathway [40, 41]. The most significant genetic association of the gene with the disease concerns the nonsynonymous SNP rs1061170 (T/C, Tyr402His). It encodes a specific CFH isoform, which modifies the CFH inhibitory function and induces the deregulation of the complement system. In particular, the homozygous CC genotype results in an increased activation of the complement cascade and has been associated with a higher risk for AMD development and progression [92, 93]. In fact, subjects who are <95 years old and carry the C variant have a 48% risk of developing AMD, as opposed to the 22% risk for noncarriers [94]. The rs1061170 SNP association with AMD was also evaluated in the Italian population to report a *P value* = 1.1∗10^−13^ and an OR = 13.06 (95% CI = 6.27–27.19) for the CC genotype [95]. Overall, these data suggest the rs1061170 SNP as one of the main genetic risk factors for AMD [92].

In 2013, several studies associated the variants in* C2*,* CFB*,* C3*,* C9*, and* CFI* genes with the disease [96].* C2* (chromosome 6p21.33, rs9332739, rs547154) and* CFB* (chromosome 6p21.33, rs4151667 and rs641153) common variations cause nonsynonymous amino acid changes [97]. In particular, rs9332739 (G/C) results in a Glu318Asp amino acid change, rs641153 (G/A) generates a Glu32Arg amino acid change, and rs4151667 (T/A) induces a Leu304His amino acid change. The remaining* C2* SNP (rs547154, G/T) is an intronic variation and does not produce any protein sequence changes. The* C2* and* CFB* genes, which encode complement component 2 and complement factor B, have been associated with a higher AMD risk [98, 99].

The* C3* gene (chromosome 19p13.3-p13.2) codes for C3 protein, which plays a pivotal role in the complement pathway via the interaction with CFH. In particular, C3 is proteolytically cleaved into C3a and C3b. Both proteins are important mediators of inflammation, with C3b acting as an opsonin (responsible for the opsonisation of microorganisms) and C3a as an essential supervisor of cytokine and chemokine expression. Two susceptibility SNPs have been described in the* C3* gene: rs147859257 (G/T, Lys155Gln) and rs2230199 (C/G, Gly102Arg) [99, 100].

The* C9* gene (chromosome 5p13.1) encodes the homonymous protein, which is involved in the final step of the complement system, via the formation of membrane attack complex (MAC). This complex is responsible for microorganism membrane disruption. In 2013, a rare variant of the* C9* gene, rs34882957 (A/G, Pro167Ser), was found to be significantly associated with AMD progression. In the same study, a variant (rs4698775, G/T) of the* CFI* gene (chromosome 4q25) was correlated with AMD development. It encodes complement factor 1, which is an essential protease implicated in the regulation of the complement cascade [96].

Given these data, a number of polymorphisms mapped on different complement genes proved to be involved in AMD pathogenesis, although* CFH* remains the main risk locus.

### 3.3. The Implication of* ARMS2* in AMD Susceptibility

The* ARMS2* gene (also known as* LOC387715*) is located on the 10q26.16 chromosome and codes for the age-related maculopathy susceptibility protein, whose function remains unknown [101–103].

Although the involvement of ARMS2 protein in AMD pathogenesis has to be yet clarified, several genetic studies described a number of alterations in the* ARMS2* gene that are strongly associated with the disease. On this subject, Fritsche et al. first identified a deletion/insertion (indel) polymorphism (composed of a 443 bp deletion followed by 54 bp insertion, del443ins54) at the 3′UTR of* ARMS2* in 2008 [47]. A molecular study demonstrated that the del443ins54 generates an unstable mRNA transcript that is subject to rapid degradation, which compromises proper protein translation [104]. The genotyping analysis of the del443ins54 polymorphism in the Italian population demonstrated a significant association with AMD susceptibility (*P value* = 2.5∗10^−16^, OR = 20.61, 95% CI = 8.83–48.11 in the presence of homozygosity for the del443ins54) [95].

Successive case/control studies and GWAS (genome-wide association studies) associated the* ARMS2* single nucleotide polymorphism, rs10490924 (G/T), with the disease [48]. The SNP is responsible for the nonsynonymous amino acid change Ala69Ser, which increases the risk for developing AMD in individuals homozygous for the T allele by 7.6-fold compared to heterozygotes. Interestingly, the del443ins54 polymorphism and the rs10490924 SNP have been found to be in strong linkage disequilibrium, which simplifies the simultaneous genetic and molecular characterization of the two risk variants in the 10q26.13 locus [99].

The presence of risk variants in the 10q26.13 locus has been further correlated with an elevated level of C-reactive protein (CRP) in subjects with no evidence of AMD compared to individuals carrying the wild-type genotypes [105]. The relationship observed between* ARMS2* risk variants and high CRP serum levels suggests the potential involvement of gene polymorphisms in inflammation and perhaps in AMD development and progression [106].

Supporting this hypothesis, an expression study carried out on* ARMS2* mRNA further described a possible relationship between the gene and some proinflammatory molecules (IL-6, IL-8, TNF-*α*, C3, and C5). This relationship suggests a potential AMD pathogenesis mechanism [72, 107]. However, further studies are necessary to clarify the role of ARMS2 protein and its involvement in disease development.

### 3.4. Inflammasome

The NLRP3 inflammasome complex is a protein complex that consists of NLRP3 (NALP3), PYCARD, and caspase-1. It can be activated by microorganisms, specific* non-self*-pathogens, or danger-associated structural patterns (PAMPs and DAMPs), and it primarily induces potent proinflammatory molecules, such as IL-1*β* and IL-18 [108, 109]. Recent studies connected NLRP3 inflammasome activity to various complex diseases, such as Alzheimer's disease, atherosclerosis, and AMD [110, 111]. Moreover, the expression of the NLRP3 inflammasome in the RPE cells and the subsequent IL-1*β* release and activation implicate it in AMD pathogenesis [112–115]. However, while molecular studies have investigated the relationship between AMD and the inflammasome, data concerning their genetic features are lacking.

## 4. Conclusions

Age-related macular degeneration (AMD) represents one of the most sight-threatening diseases in developed countries. The prevalence of AMD is 2.1% in individuals aged 40–49 years and increases dramatically to 35% in subjects aged >80 years [116]. The clinical course of the disease is highly variable and ranges from patients progressing to later AMD forms with little clinical evidence to patients whose clinical conditions may be stable for decades. Specifically, approximately 10–20% of patients with nonexudative AMD may progress to the exudative form if untreated [117].

An extensive study focused on the interplay between the genetic background and environmental factors involved in AMD aetiopathogenesis. Among the different risk factors (ageing, ethnicity, gender, hypertension, genetics, diet, and sunlight exposure), age represents one of the major contributors in the development of the disease. In fact, the common ageing processes are known to be responsible for structural and blood flow changes in the eye, thereby leading to the alteration and degeneration of the* macula lutea*. In particular, a progressive increase in the number of drusen results in the structural alteration of the macula and, subsequently, in the persistent induction of immune and inflammatory events. However, these age-related effects are not always responsible for AMD development and cannot be considered as the only triggering factors, which highlights the crucial role of genetics in the development and progression of the disease. In fact, 50–60% of the disease aetiology can be attributed to the genetic variations in the* CFH*,* ARMS2*, and* IL-8* genes, which encode complement factor H, age-related maculopathy susceptibility protein 2, and interleukin-8, respectively. Conversely, molecular evidence recently implicated the NLRP3 inflammasome as a potential inflammatory contributor in AMD pathogenesis, although genetic data on this subject are not available. The large majority of genetic studies performed to date have highlighted immunity and inflammation as the main characters that play a pivotal role in the pathogenesis of AMD.

In conclusion, several genetic and molecular studies have attempted to describe the pathogenetic pathways that are potentially involved in AMD. The age of the individual, other risk factors, and the genetic assessment of the individual are well known to likely affect the clinical development and progression of AMD. Future efforts should attempt to improve the genetic and epigenetic characterization of the inflammatory pathways in AMD in order to discover both innate and age-related predictive disease biomarkers as well as potential therapeutic targets.

## Figures and Tables

**Table 1:** Candidate genes involved in AMD pathogenesis, following GWAS.

Gene	Locus	SNP
*C2-CFB *	6p21.33	rs429608 A/G
*C3 *	19p13.3	rs2230199 C/G
*TIMP3 *	22q12.3	rs5749482 C/G
*APOE *	19q13.2	rs4420638 G/A
*CETP *	16q21	rs1864163 G/A
*VEGFA *	6p21.1	rs943080 C/T
*TNFRSF10A *	8p21.3	rs13278062 G/T
*LIPC *	15q21	rs920915 C/G
*CFI *	4q25	rs4698775 G/T
*COL10A1 *	6q21	rs3812111 A/T
*COL8A1 *	3q12.1	rs13081855 G/T
*IER3 *	6p21.3	rs3130783 A/G
*SLC16A8 *	22q13.1	rs8135665 C/T
*TGFBR1 *	9q22	rs334353 T/G
*RAD51B *	14q23	rs8017304 A/G
*ADAMTS9 *	3p14.1	rs6795735 C/T
*B3GALTL *	13q12.3	rs9542236 C/T

**Table 2:** AMD genes involved in the inflammatory pathway.

Gene	Locus	Function
*IL-1 *	2q14	Cytokine
*IL-6 *	7p15.3	Cytokine
*IL-8 *	4q12-q13	Chemokine
*CCL3 *	17q11.2-q12	Chemokine
*CCL4 *	17q11.2-q12	Chemokine
*CCR3 *	3p21.3	Chemokine receptor
*CFH *	1q31	Complement factor H
*C2 *	6p21.33	Complement component 2
*CFB *	6p21.33	Complement factor B
*C3 *	19p13.3-p13.2	Complement component 3
*C9 *	5p13.1	Complement component 9
*CFI *	4q25	Complement factor 1
*ARMS2 *	10q26.16	Possible activation of proinflammatory molecules
*NLRP3 *	1q44	Inflammasome
